# The genome sequence of the Ragwort Fly, *Sphenella marginata *(Fallén, 1814)

**DOI:** 10.12688/wellcomeopenres.19841.1

**Published:** 2023-09-01

**Authors:** Steven Falk, Ryan Mitchell, Xavier Richard Badham, University of Oxford and Wytham Woods Genome Acquisition Lab, Darwin Tree of Life Barcoding collective, Wellcome Sanger Institute Tree of Life programme, Wellcome Sanger Institute Scientific Operations: DNA Pipelines collective, Tree of Life Core Informatics collective, Darwin Tree of Life Consortium

**Affiliations:** 1 Independent researcher, Kenilworth, England, UK; 2 Independent researcher, Sligo, Ireland; 3 University of Aberdeen, Aberdeen, Scotland, UK; 4 Queen's University Belfast, Belfast, Northern Ireland, UK

**Keywords:** Sphenella marginata, Ragwort Fly, genome sequence, chromosomal, Diptera

## Abstract

We present a genome assembly from an individual female *Sphenella marginata* (the Ragwort Fly; Arthropoda; Insecta; Diptera; Tephritidae). The genome sequence is 595.2 megabases in span. Most of the assembly is scaffolded into 6 chromosomal pseudomolecules. The mitochondrial genome has also been assembled and is 16.82 kilobases in length.

## Species taxonomy

Eukaryota; Metazoa; Eumetazoa; Bilateria; Protostomia; Ecdysozoa; Panarthropoda; Arthropoda; Mandibulata; Pancrustacea; Hexapoda; Insecta; Dicondylia; Pterygota; Neoptera; Endopterygota; Diptera; Brachycera; Muscomorpha; Eremoneura; Cyclorrhapha; Schizophora; Acalyptratae; Tephritoidea; Tephritidae; Tephritinae; Tephritini; *Sphenella* genus group; *Sphenella*; *Sphenella marginata* ( Fallén, 1814) (NCBI:txid594017).

## Background

*Sphenella marginata* ( Fallén, 1814) is a species of fly in the family Tephritidae. This species is found throughout the Palearctic region. *Sphenella marginata* likely has the widest natural distribution of any Tephritid ( White, 1988). In Britain, it is found towards the south of England and Wales with only rare occurrence further north. In Ireland, it has been officially recorded in Northern Ireland only, but it is likely found further south, as *S. marginata* distribution follows the distribution of ragworts and groundsels. No IUCN Global Red List category has been published as of the date of this publication.

Colloquially *S. marginata* is known as the Ragwort Fly. This is due to the larvae being known to feed within the capitulum of numerous *Senecio* plants ( Schmidl, 1972), otherwise known as ragworts and groundsels. The activity of *S.marginata* larvae causes galls, where flower heads swell at the base and bracts enlarge. Usually the flower head will contain only one white-coloured larvae or brown puparium. The species is diurnal.

Adults have long geniculate mouthparts, indicating the possibility of nectar feeding ( White, 1988). The frons and legs are yellow-orange, the thorax has an earthy brown colour, whilst the abdomen has a dark brown colour. The eyes of adult flies are verdant green. The costal margins of the forewings are spotted brown, with a full dark brown bar across the apex and where the abdomen begins, whilst the hindwings are clear.

The genome of the Ragwort Fly, *Sphenella marginata*, was sequenced as part of the Darwin Tree of Life Project, a collaborative effort to sequence all named eukaryotic species in the Atlantic Archipelago of Britain and Ireland.

## Genome sequence report

**Figure 1.**
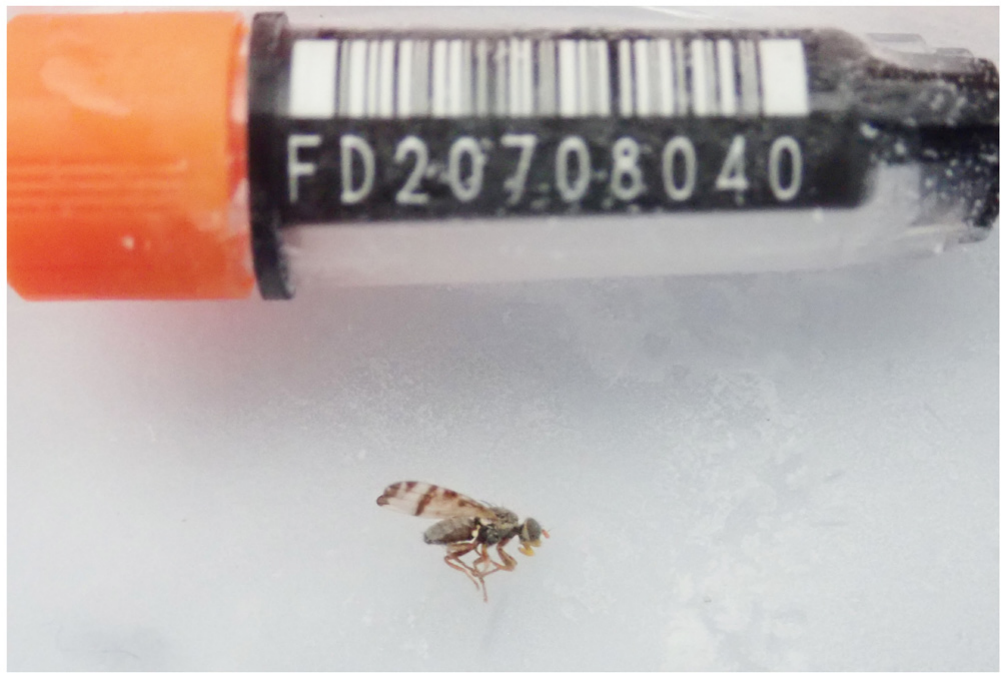
Photograph of the *Sphenella marginata* (idSphMarg1) specimen used for genome sequencing.

The genome was sequenced from one *Sphenella marginata* ( Figure 1) collected from Wytham Woods, Oxfordshire, UK (51.77, –1.33). A total of 31-fold coverage in Pacific Biosciences single-molecule HiFi long reads was generated. Primary assembly contigs were scaffolded with chromosome conformation Hi-C data. Manual assembly curation corrected 318 missing joins or mis-joins and removed 22 haplotypic duplications, reducing the assembly length by 0.53% and the scaffold number by 62.38%, and increasing the scaffold N50 by 46.76%.

**Figure 2.**
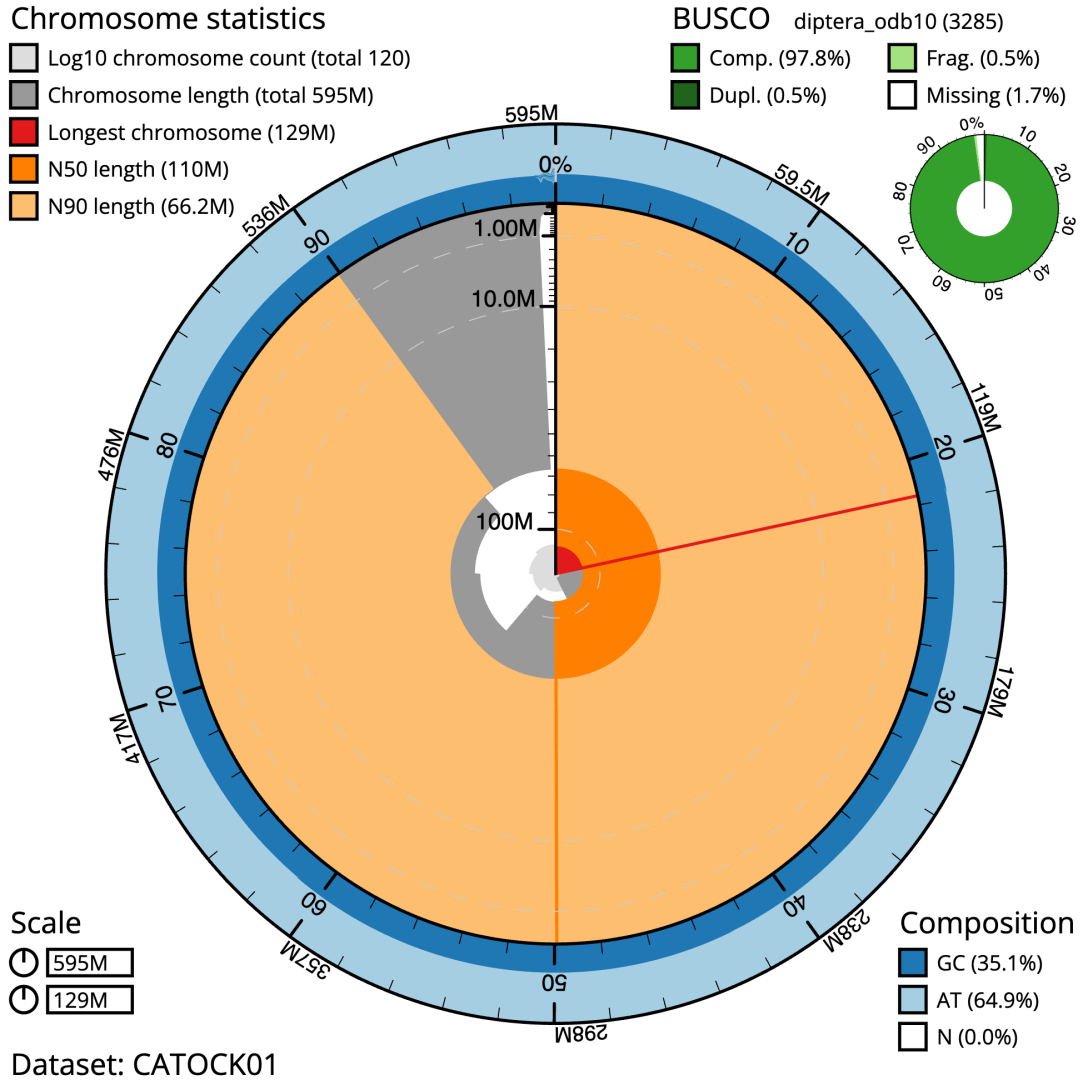
Genome assembly of *Sphenella marginata*, idSphMarg1.1: metrics.. The BlobToolKit Snailplot shows N50 metrics and BUSCO gene completeness. The main plot is divided into 1,000 size-ordered bins around the circumference with each bin representing 0.1% of the 595,252,386 bp assembly. The distribution of scaffold lengths is shown in dark grey with the plot radius scaled to the longest scaffold present in the assembly (129,039,367 bp, shown in red). Orange and pale-orange arcs show the N50 and N90 scaffold lengths (109,573,589 and 66,161,324 bp), respectively. The pale grey spiral shows the cumulative scaffold count on a log scale with white scale lines showing successive orders of magnitude. The blue and pale-blue area around the outside of the plot shows the distribution of GC, AT and N percentages in the same bins as the inner plot. A summary of complete, fragmented, duplicated and missing BUSCO genes in the diptera_odb10 set is shown in the top right. An interactive version of this figure is available at https://blobtoolkit.genomehubs.org/view/idSphMarg1.1/dataset/CATOCK01/snail.

The final assembly has a total length of 595.2 Mb in 119 sequence scaffolds with a scaffold N50 of 109.6 Mb ( Table 1). Most (99.36%) of the assembly sequence was assigned to 6 chromosomal-level scaffolds. Chromosome-scale scaffolds confirmed by the Hi-C data are named in order of size ( Figure 2– Figure 5; Table 2). The sole sex chromosome in this assembly is chromosome 1, which has approximately two-thirds coverage compared to the autosomes. There is no evidence of a Y chromosome, thus the data indicates that this species has homomorphic sex chromosomes. While not fully phased, the assembly deposited is of one haplotype. Contigs corresponding to the second haplotype have also been deposited. The mitochondrial genome was also assembled and can be found as a contig within the multifasta file of the genome submission.

**Table 1.** Genome data for *Sphenella marginata*, idSphMarg1.1.

Project accession data		
Assembly identifier	idSphMarg1.1	
Species	*Sphenella marginata*	
Specimen	idSphMarg1	
NCBI taxonomy ID	594017	
BioProject	PRJEB59087	
BioSample ID	SAMEA7746749	
Isolate information	idSphMarg1, whole organism (DNA sequencing) idSphMarg2, whole organism (Hi-C scaffolding and RNA sequencing)	
Assembly metrics *		*Benchmark*
Consensus quality (QV)	57.4	*≥ 50*
*k*-mer completeness	99.99%	*≥ 95%*
BUSCO **	C:97.8%[S:97.2%,D:0.5%], F:0.5%,M:1.7%,n:3,285	*C ≥ 95%*
Percentage of assembly mapped to chromosomes	99.36%	*≥ 95%*
Sex chromosomes	Chromosome 1 (Not assigned)	*localised homologous pairs*
Organelles	Mitochondrial genome assembled.	*complete single alleles*
Raw data accessions		
PacificBiosciences SEQUEL II	ERR10798435	
Hi-C Illumina	ERR10802459	
PolyA RNA-Seq Illumina	ERR11641120	
Genome assembly		
Assembly accession	GCA_951509765.1	
*Accession of alternate haplotype*	GCA_951509705.1	
Span (Mb)	595.2	
Number of contigs	1389	
Contig N50 length (Mb)	0.8	
Number of scaffolds	119	
Scaffold N50 length (Mb)	109.6	
Longest scaffold (Mb)	129.0	

* Assembly metric benchmarks are adapted from column VGP-2020 of “Table 1: Proposed standards and metrics for defining genome assembly quality” from ( Rhie *et al.*, 2021). ** BUSCO scores based on the diptera_odb10 BUSCO set using v5.3.2. C = complete [S = single copy, D = duplicated], F = fragmented, M = missing, n = number of orthologues in comparison. A full set of BUSCO scores is available at https://blobtoolkit.genomehubs.org/view/idSphMarg1.1/dataset/CATOCK01/busco.

**Figure 3.**
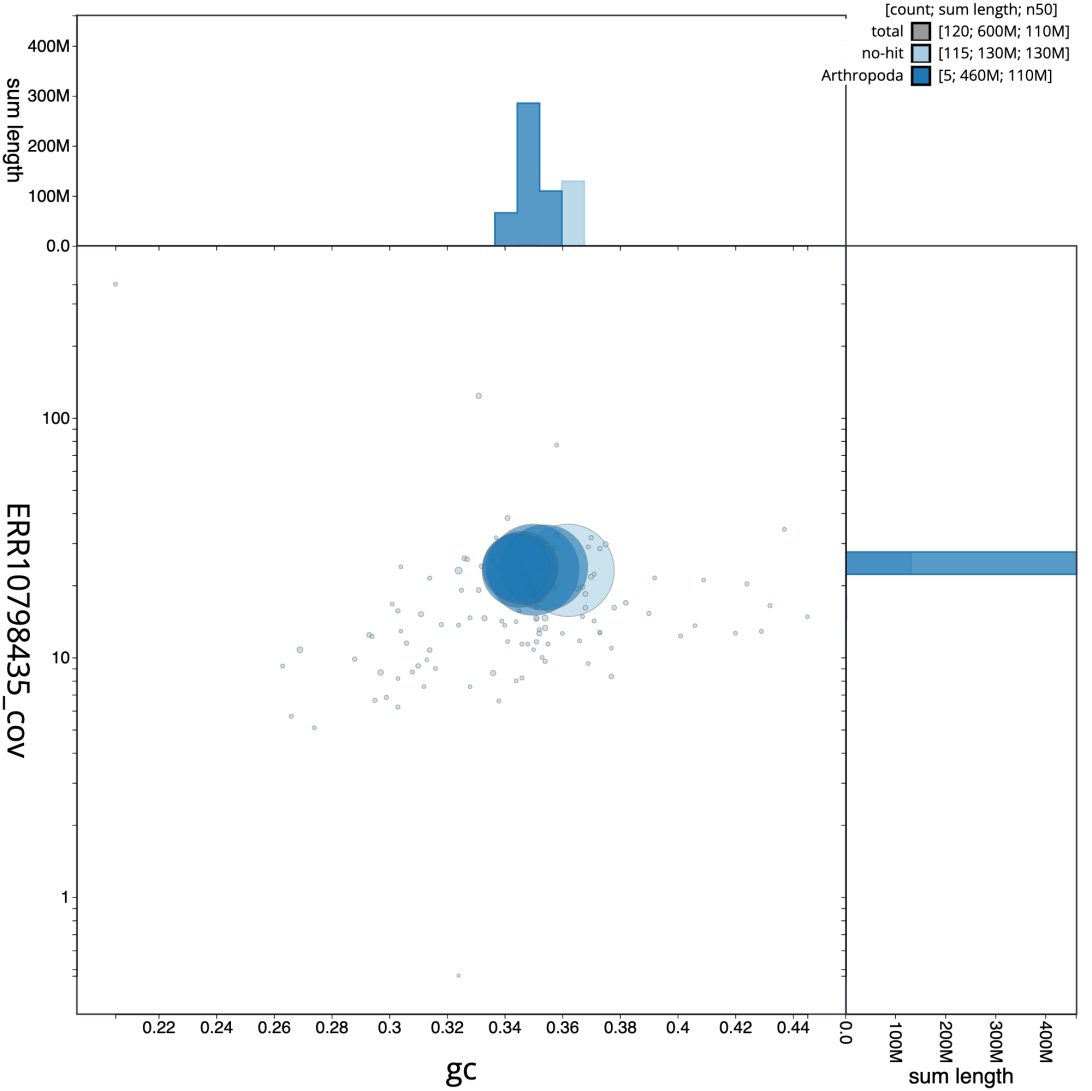
Genome assembly of *Sphenella marginata*, idSphMarg1.1: BlobToolKit GC-coverage plot. Scaffolds are coloured by phylum. Circles are sized in proportion to scaffold length. Histograms show the distribution of scaffold length sum along each axis. An interactive version of this figure is available at https://blobtoolkit.genomehubs.org/view/idSphMarg1.1/dataset/CATOCK01/blob.

**Figure 4.**
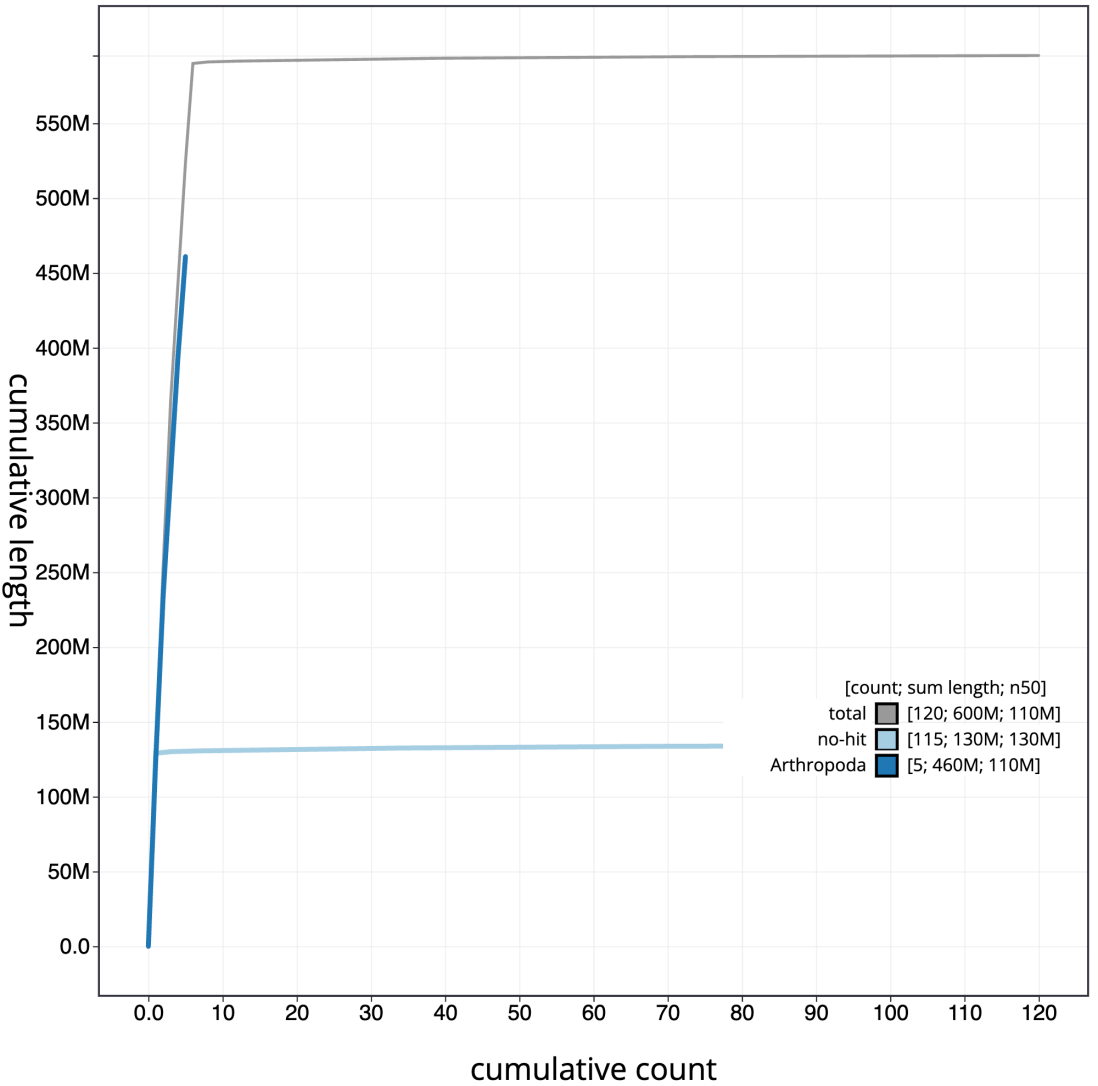
Genome assembly of *Sphenella marginata*, idSphMarg1.1: BlobToolKit cumulative sequence plot. The grey line shows cumulative length for all scaffolds. Coloured lines show cumulative lengths of scaffolds assigned to each phylum using the buscogenes taxrule. An interactive version of this figure is available at https://blobtoolkit.genomehubs.org/view/idSphMarg1.1/dataset/CATOCK01/cumulative.

**Figure 5.**
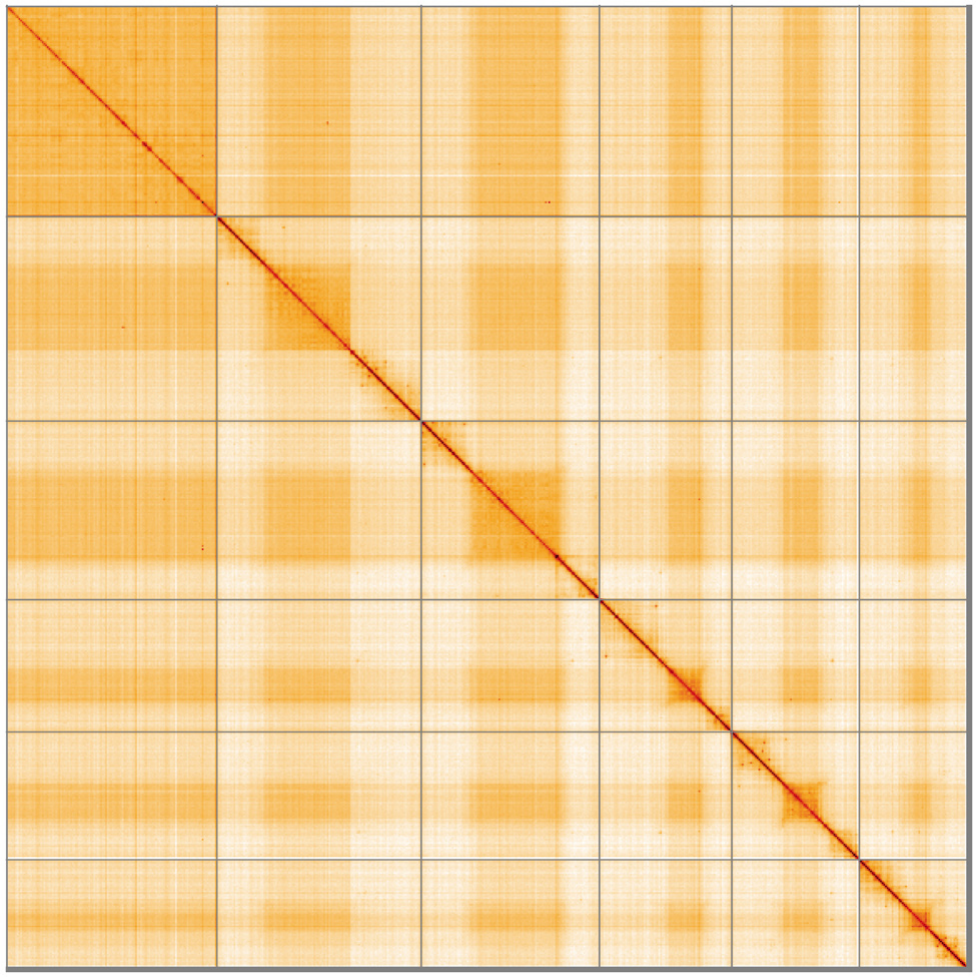
Genome assembly of *Sphenella marginata*, idSphMarg1.1: Hi-C contact map of the idSphMarg1.1 assembly, visualised using HiGlass. Chromosomes are shown in order of size from left to right and top to bottom. An interactive version of this figure may be viewed at https://genome-note-higlass.tol.sanger.ac.uk/l/?d=GGd34c4iSHOvGtSr3Yyt6Q.

**Table 2.** Chromosomal pseudomolecules in the genome assembly of *Sphenella marginata*, idSphMarg1.

INSDC accession	Chromosome	Length (Mb)	GC%
OX608078.1	1	129.04	36.0
OX608079.1	2	125.53	35.0
OX608080.1	3	109.57	35.5
OX608081.1	4	81.29	34.5
OX608082.1	5	78.35	34.5
OX608083.1	6	66.16	34.5
OX608084.1	MT	0.02	20.5

The estimated Quality Value (QV) of the final assembly is 57.4 with *k*-mer completeness of 100%, and the assembly has a BUSCO v5.3.2 completeness of 97.8% (single = 97.2%, duplicated = 0.5%), using the diptera_odb10 reference set ( *n* = 3,285).

Metadata for specimens, spectral estimates, sequencing runs, contaminants and pre-curation assembly statistics can be found at https://links.tol.sanger.ac.uk/species/594017.

## Methods

### Sample acquisition and nucleic acid extraction

The *Sphenella marginata* specimen (specimen ID Ox000842, individual idSphMarg1) used for sequencing was collected from Wytham Woods, Oxfordshire (biological vice-county Berkshire), UK (latitude 51.77, longitude –1.33) on 2020-08-04 by netting. The specimen was collected and identified by Steven Falk (independent researcher). The specimen used to generate Hi-C data and for RNA sequencing (specimen ID Ox001853, individual idSphMarg2) was collected by Ryan Mitchell (independent researcher) from the same location on 2021-08-21. Both specimens were preserved on dry ice.

DNA was extracted at the Tree of Life laboratory, Wellcome Sanger Institute (WSI). The idSphMarg1 sample was weighed and dissected on dry ice. Tissue from the whole organism was disrupted using a Nippi Powermasher fitted with a BioMasher pestle. High molecular weight (HMW) DNA was extracted using the Qiagen MagAttract HMW DNA extraction kit. HMW DNA was sheared into an average fragment size of 12–20 kb in a Megaruptor 3 system with speed setting 30. Sheared DNA was purified by solid-phase reversible immobilisation using AMPure PB beads with a 1.8X ratio of beads to sample to remove the shorter fragments and concentrate the DNA sample. The concentration of the sheared and purified DNA was assessed using a Nanodrop spectrophotometer and Qubit Fluorometer and Qubit dsDNA High Sensitivity Assay kit. Fragment size distribution was evaluated by running the sample on the FemtoPulse system.

RNA was extracted from tissue of idSphMarg2 in the Tree of Life Laboratory at the WSI using TRIzol, according to the manufacturer’s instructions. RNA was then eluted in 50 μl RNAse-free water and its concentration assessed using a Nanodrop spectrophotometer and Qubit Fluorometer using the Qubit RNA Broad-Range (BR) Assay kit. Analysis of the integrity of the RNA was done using Agilent RNA 6000 Pico Kit and Eukaryotic Total RNA assay.

### Sequencing

Pacific Biosciences HiFi circular consensus DNA sequencing libraries were constructed according to the manufacturers’ instructions. Poly(A) RNA-Seq libraries were constructed using the NEB Ultra II RNA Library Prep kit. DNA and RNA sequencing was performed by the Scientific Operations core at the WSI on Pacific Biosciences SEQUEL II (HiFi) and Illumina NovaSeq 6000 (RNA-Seq) instruments. Hi-C data were also generated from tissue of idSphMarg2 using the Arima2 kit and sequenced on the Illumina NovaSeq 6000 instrument.

### Genome assembly, curation and evaluation

Assembly was carried out with Hifiasm ( Cheng *et al.*, 2021) and haplotypic duplication was identified and removed with purge_dups ( Guan *et al.*, 2020). The assembly was then scaffolded with Hi-C data ( Rao *et al.*, 2014) using YaHS ( Zhou *et al.*, 2023). The assembly was checked for contamination and corrected as described previously ( Howe *et al.*, 2021). Manual curation was performed using HiGlass ( Kerpedjiev *et al.*, 2018) and Pretext ( Harry, 2022). The mitochondrial genome was assembled using MitoHiFi ( Uliano-Silva *et al.*, 2023), which runs MitoFinder ( Allio *et al.*, 2020) or MITOS ( Bernt *et al.*, 2013) and uses these annotations to select the final mitochondrial contig and to ensure the general quality of the sequence.

A Hi-C map for the final assembly was produced using bwa-mem2 ( Vasimuddin *et al.*, 2019) in the Cooler file format ( Abdennur & Mirny, 2020). To assess the assembly metrics, the *k*-mer completeness and QV consensus quality values were calculated in Merqury ( Rhie *et al.*, 2020). This work was done using Nextflow ( Di Tommaso *et al.*, 2017) DSL2 pipelines “sanger-tol/readmapping” ( Surana *et al.*, 2023a) and “sanger-tol/genomenote” ( Surana *et al.*, 2023b). The genome was analysed within the BlobToolKit environment ( Challis *et al.*, 2020) and BUSCO scores ( Manni *et al.*, 2021; Simão *et al.*, 2015) were calculated.

Table 3 contains a list of relevant software tool versions and sources.

**Table 3.** Software tools: versions and sources.

Software tool	Version	Source
BlobToolKit	4.1.7	https://github.com/blobtoolkit/ blobtoolkit
BUSCO	5.3.2	https://gitlab.com/ezlab/busco
Hifiasm	0.16.1-r375	https://github.com/chhylp123/ hifiasm
HiGlass	1.11.6	https://github.com/higlass/higlass
Merqury	MerquryFK	https://github.com/thegenemyers/ MERQURY.FK
MitoHiFi	2	https://github.com/marcelauliano/ MitoHiFi
PretextView	0.2	https://github.com/wtsi-hpag/ PretextView
purge_dups	1.2.3	https://github.com/dfguan/purge_ dups
sanger-tol/ genomenote	v1.0	https://github.com/sanger-tol/ genomenote
sanger-tol/ readmapping	1.1.0	https://github.com/sanger-tol/ readmapping/tree/1.1.0
YaHS	1.2a	https://github.com/c-zhou/yahs

### Wellcome Sanger Institute – Legal and Governance

The materials that have contributed to this genome note have been supplied by a Darwin Tree of Life Partner. The submission of materials by a Darwin Tree of Life Partner is subject to the **‘Darwin Tree of Life Project Sampling Code of Practice’**, which can be found in full on the Darwin Tree of Life website here. By agreeing with and signing up to the Sampling Code of Practice, the Darwin Tree of Life Partner agrees they will meet the legal and ethical requirements and standards set out within this document in respect of all samples acquired for, and supplied to, the Darwin Tree of Life Project.

Further, the Wellcome Sanger Institute employs a process whereby due diligence is carried out proportionate to the nature of the materials themselves, and the circumstances under which they have been/are to be collected and provided for use. The purpose of this is to address and mitigate any potential legal and/or ethical implications of receipt and use of the materials as part of the research project, and to ensure that in doing so we align with best practice wherever possible. The overarching areas of consideration are:

•   Ethical review of provenance and sourcing of the material

•   Legality of collection, transfer and use (national and international) 

Each transfer of samples is further undertaken according to a Research Collaboration Agreement or Material Transfer Agreement entered into by the Darwin Tree of Life Partner, Genome Research Limited (operating as the Wellcome Sanger Institute), and in some circumstances other Darwin Tree of Life collaborators.

## Data Availability

European Nucleotide Archive: *Sphenella marginata* (ragwort fly). Accession number PRJEB59087; https://identifiers.org/ena.embl/PRJEB59087. ( Wellcome Sanger Institute, 2022) The genome sequence is released openly for reuse. The *Sphenella marginata* genome sequencing initiative is part of the Darwin Tree of Life (DToL) project. All raw sequence data and the assembly have been deposited in INSDC databases. The genome will be annotated using available RNA-Seq data and presented through the Ensembl pipeline at the European Bioinformatics Institute. Raw data and assembly accession identifiers are reported in Table 1.

## Author information

Members of the University of Oxford and Wytham Woods Genome Acquisition Lab are listed here: https://doi.org/10.5281/zenodo.4789928.

Members of the Darwin Tree of Life Barcoding collective are listed here: https://doi.org/10.5281/zenodo.4893703.

Members of the Wellcome Sanger Institute Tree of Life programme are listed here: https://doi.org/10.5281/zenodo.4783585.

Members of Wellcome Sanger Institute Scientific Operations: DNA Pipelines collective are listed here: https://doi.org/10.5281/zenodo.4790455.

Members of the Tree of Life Core Informatics collective are listed here: https://doi.org/10.5281/zenodo.5013541.

Members of the Darwin Tree of Life Consortium are listed here: https://doi.org/10.5281/zenodo.4783558.
