# Supplementary material for: The genome sequence of the Ragwort Fly, Sphenella marginata (Fallén, 1814)
Source: Wellcome Open Res. 2023 Sep 1;8:383. [Version 1] doi: 10.12688/wellcomeopenres.19841.1 (PMC11344206; doi:10.12688/wellcomeopenres.19841.1)
Supplement: Peer review history

# Reviewer report: Reviewer response for version 1

**DOI:** 10.21956/wellcomeopenres.21973.r93331

**Published:** 2024-08-23

**Authors:** Madeleine Aase-Remedios

**Affiliations:** 1 Durham University, Durham, England, UK

The authors present a high-quality chromosome-level genome assembly for the ragwort fly *Sphenella marginata*, using state-of-the-art methods. While rationale is not explicitly given, it is obviously part of the larger DToL project's aims.

 Overall, the data note is sufficient for its purposes, though there are a few suggestions that would improve its reproducibility and utility as a resource.

- The data note explains the source of collection of the specimens but does not explicitly mention identifying marks that distinguish this species from other similar flies, though this information can presumably be found in other sources, which the authors could reference.
- Though RNA was sequenced, the annotation is not included, hindering the utility of this resource. I would prefer annotations to be published alongside the genome assemblies, but I understand the benefit of publishing the genome assembly sooner.
- The method section contains references and links to pipelines, packages, and versions, but does not include all details to make this fully replicable (on parameters used, etc.).

Are sufficient details of methods and materials provided to allow replication by others?

Yes

Is the rationale for creating the dataset(s) clearly described?

Yes

Are the datasets clearly presented in a useable and accessible format?

Yes

Are the protocols appropriate and is the work technically sound?

Yes

Reviewer Expertise:

Arachnid development, genomics, and transcriptomics

I confirm that I have read this submission and believe that I have an appropriate level of expertise to confirm that it is of an acceptable scientific standard.

# Reviewer report: Reviewer response for version 1

**DOI:** 10.21956/wellcomeopenres.21973.r90271

**Published:** 2024-08-12

**Authors:** Claus Vogl

**Affiliations:** 1 Vetmeduni Vienna, Wien, Austria

**Detail: **

- The singular of "larvae" is "larva", hence it must be "one ... larva" and not "one ... larvae".
- Lab and bioinformatics methods and analyses are state of the art. The writing is straightforward and clear. 

My only substantial comment revolves around the sex of the specimens and the nature of the sex chromosome: I could not find any indication with the sex of the sequenced specimens, and it is not clear how the authors determined that chromosome 1 is actually the sex chromosome. I would urge the authors to add this information.

Are sufficient details of methods and materials provided to allow replication by others?

Yes

Is the rationale for creating the dataset(s) clearly described?

Yes

Are the datasets clearly presented in a useable and accessible format?

Yes

Are the protocols appropriate and is the work technically sound?

Yes

Reviewer Expertise:

population genetics, population genomics, bioinformatcs

I confirm that I have read this submission and believe that I have an appropriate level of expertise to confirm that it is of an acceptable scientific standard.
